# A Multifunctional Cementitious Composite for Pavement Subgrade

**DOI:** 10.3390/ma17030621

**Published:** 2024-01-27

**Authors:** Mohammad Jawed Roshan, Mohammadmahdi Abedi, António Gomes Correia, Raul Fangueiro, Paulo Mateus Mendes

**Affiliations:** 1 Department of Civil Engineering, ISISE, ARISE, University of Minho, Campus de Azurém, 4800-058 Guimarães, Portugal; jroshan2020@gmail.com (M.J.R.); mohammadmehdi.abedi@gmail.com (M.A.); 2 Centre for Textile Science and Technology, Department of Textile Engineering, University of Minho, Campus de Azurém, 4800-058 Guimarães, Portugal; 3 Center for Microelectromechanical Systems (CMEMS-UMinho), Department of Industrial Electronics, University of Minho, Campus de Azurém, 4800-058 Guimarães, Portugal; pmendes@dei.uminho.pt

**Keywords:** self-sensing cementitious composite, MWCNT/GNP, stress/strain-sensing, resilient modulus, damage detection, electrochemical impedance spectroscopy (EIS)

## Abstract

Premature failure and degradation of layers are the main problems for transportation infrastructure. Addressing these issues necessitates implementing structural health monitoring (SHM) for pavement construction layers. To this end, this research investigated the stress/strain and damage detection capabilities of a self-sensing cementitious composite developed for potential utilization in the construction of an intelligent subgrade layer. The prepared self-sensing cementitious composite consisted of 10% cement and hybrid conductive fillers, including multiwalled carbon nanotubes (MWCNTs) and graphene nanoplatelets (GNPs) in sand. Initial findings reveal that the electrical resistivity of the composite is significantly affected by the concentration of MWCNTs/GNPs, with a minimum concentration of more than 0.5% needed to achieve a responsive cementitious composite. Moreover, the piezoresistive analysis indicates that an increase in the concentration of MWCNTs/GNPs and stress levels leads to an improvement in the stress/strain-sensing performance. When the self-sensing cementitious composite is subjected to equivalent stress levels, variations in the fractional changes in resistivity (FCR) exhibit an increasing trend with decreasing resilient modulus, stemming from a decrease in stiffness due to the increased concentration of MWCNTs/GNPs. Additionally, the electrochemical impedance spectroscopy (EIS) analysis demonstrates a contraction for the Nyquist plots under compressive ramp loading prior to failure, followed by the expansion of these curves post-failure. Scanning electron microscopy (SEM) images visually showcase the bridging effects of MWCNTs and the filling effects of GNPs within the composite structure.

## 1. Introduction

Transportation infrastructure, particularly road networks, plays a pivotal role in facilitating societal connectivity and the efficient movement of passengers and goods, thereby contributing to economic development [1]. Consequently, it is imperative to maintain the various layers of road infrastructure to ensure smooth and convenient travel between different regions. To achieve this objective, substantial financial resources are allocated by authorities for the construction and maintenance of road networks. Nonetheless, challenges persist in the form of road pavement defects and transportation infrastructure failures, including issues such as rutting, cracking, reduced durability, slope instability, and excessive permanent strain accumulation [2,3,4]. These failures can be attributed to various factors, such as environmental conditions, groundwater levels, and excessive loading [5,6,7,8,9]. Among these factors, environmental elements—including freeze–thaw cycles, wet–dry cycles, temperature variations, and rainfall infiltration—play a pivotal role in causing premature failures in transportation infrastructure. For example, Decky et al. [9] highlighted the significant impact of temperature on the performance of rigid pavement, advocating for the incorporation of temperature effects into standard regulations. In a separate study, Bilodeau et al. [5] observed an increase in the degree of saturation and a decrease in the dry density of unbound granular material (UGM) during freeze–thaw cycles, leading to increased permanent deformation.

Furthermore, it is important to note that pavement layers are interconnected in such a way that the failure of one layer can lead to the deterioration of other layers. Among these layers, the subgrade layer, characterized by its weak soil properties, stands out as a significant factor contributing to the failure of the upper layers [10,11]. Therefore, it is crucial to ensure that the subgrade soil, serving as the foundation for the upper layers, is in optimal condition. To address this issue, various techniques have been employed to enhance the performance of subgrade layers, with cement stabilization being a commonly employed method [12,13,14]. Nevertheless, prior research has predominantly focused on the mechanical aspects of subgrade performance when using conventional cement stabilization techniques [1].

Enhancing the functionality of the subgrade layer can be achieved through the innovative utilization of self-sensing cement-stabilized sand, leading to improvements in both mechanical strength and inherent self-sensing capabilities [15]. Consequently, self-sensing cementitious composites have the capacity to autonomously monitor the subgrade layer’s condition, eliminating the need for various external sensors that have been adopted in previous studies [16,17,18,19,20,21]. The multifunctional cementitious composite serves a diverse array of purposes, including applications in structural health monitoring, stress- and strain-sensing, weigh-in-motion systems, and deicing and snow-melting [22,23,24,25]. For instance, a multifunctional cementitious composite based on graphene nanoplatelets (GNPs) and carbon nanotubes (CNTs) has been employed in structural health monitoring, as documented by Wang and Zhang (2022) [26]. Stress and strain detection has also been realized using self-sensing cementitious composites, as evidenced by previous studies [27,28,29]. Moreover, this material can be employed for deicing and snow-melting applications, which are essential in colder regions to mitigate ice formation and facilitate snow melting [30,31]. Deicing chemical agents have been extensively employed to facilitate the thawing of snow and ice, thereby enhancing safety during travel [32]. Nonetheless, the use of deicing chemicals can have detrimental effects, leading to a reduction in the mechanical strength and durability of both the pavement and the materials in adjacent layers. Consequently, adopting multifunctional cementitious composites is a viable alternative for addressing deicing and snow melting requirements [33]. For instance, multifunctional cementitious composites containing carbon fiber (CF) have been employed for deicing and snow-melting purposes, as demonstrated by [30]. All these multifaceted capabilities can be incorporated into conventional cementitious composites by introducing conductive fillers [34].

Various conductive fillers, including both metal-based and carbon-based materials, have found application in self-sensing cementitious composites [35,36]. However, it is important to acknowledge that the potential corrosion of metal-based conductive fillers can lead to a degradation of the self-sensing capabilities over time. Consequently, carbon-based conductive fillers, particularly carbon nanomaterials (CNMs), such as carbon nanotubes (CNTs), graphene nanoplatelets (GNPs), carbon black (CB), carbon fibers (CFs), and carbon nanofibers (CNFs), have gained widespread acceptance in self-sensing cementitious composites [37,38,39,40]. For instance, a study by Falara et al. examined self-sensing cementitious composites incorporating multiwalled carbon nanotubes (MWCNTs), revealing an enhanced self-sensing capability with increasing MWCNT content [41]. Nevertheless, it is worth noting that the high cost associated with SWCNTs presents a significant challenge, limiting their utilization in self-sensing cementitious composites. To address this concern, multiwalled carbon nanotubes (MWCNTs) have been introduced as an alternative in self-sensing cementitious composites [42,43]. Similarly, GNP, CF, and CNF have also been extensively investigated in prior research [44,45].

Several variables, including the concentration of conductive fillers, the type of matrix materials, the water-to-cement ratio, the sand-to-cement ratio, curing duration, moisture content, loading conditions, and the dispersion of carbon-based conductive materials, can have a substantial impact on the performance of multifunctional cementitious composites [46,47,48,49]. The effects of these influencing factors on the performance of self-sensing cementitious composites have been extensively documented in prior research. For instance, Teomete delved into the impact of moisture content [47], Guo et al. scrutinized the implications of loading conditions [46], and Sevim et al. examined the influence of conductive filler type and concentration [50].

Another intriguing topic pertains to the use of electrochemical impedance spectroscopy (EIS) to characterize cementitious composites. This analytical technique offers insights into how microstructural changes in these composites are influenced by various external factors, including moisture, the infiltration of different chemical solutions into pore spaces, the corrosion of metallic materials within the cementitious composite, the hydration process, and applied loads [51,52,53,54,55]. Since its initial application to cement paste [56], EIS has been increasingly employed to evaluate the microstructural properties of conventional cementitious composites [57,58,59,60,61]. For instance, Dong et al. investigated the carbonation process in fly ash-blended cementitious composites, observing an increase in the diameter of the semicircle in the Nyquist plots with rising carbonation [61]. In another study, Fan et al. assessed the effects of damage and self-healing in cementitious composites using EIS [62]. Nevertheless, the impedance encountered at the contact point between the electrodes and the cementitious composite can influence the appearance of the Nyquist plot, resulting in various shapes. To mitigate the impact of contact impedance, the application of a conductive medium between the electrodes and cementitious composite has been proposed [63]. In light of these considerations, it is important to note that the effects of contact impedance in self-sensing cementitious composites containing carbon-based conductive fillers may be minimal. However, in contrast to using EIS for characterizing conventional cementitious composites, there is limited application of EIS for assessing microstructural changes in self-sensing cementitious composites containing carbon derivatives [64,65,66]. To address this gap, further studies investigating the use of EIS for the characterization of self-sensing cementitious composites, with a focus on microstructural changes, are warranted.

While various types of self-sensing cementitious composites, such as self-sensing cement paste, self-sensing mortar, and self-sensing concrete, have been extensively researched [22,67,68,69], the field of self-sensing cement-stabilized sand remains relatively uncharted [15]. This distinction is attributed to the differing material composition of self-sensing cement-stabilized sand compared to conventional concrete, mortar, and cement paste. As a result, the present study is oriented towards enhancing the characteristics of a 10% cement-stabilized sand mixture by incorporating a blend of multiwalled carbon nanotubes (MWCNTs) and graphene nanoplatelets (GNPs). To achieve the objectives of this investigation, 1%, 2%, 3% and 4% MWCNTs and GNPs were integrated into a 10% cement-stabilized sand mixture. Subsequently, after the specimens were cured over a 28-day period, they were subjected to testing involving both cyclic compressive loading and monotonic ramp loading for piezoresistivity and electrochemical impedance spectroscopy (EIS) analysis, respectively. Furthermore, a microstructural analysis was conducted to gain deeper insight into the impact of MWCNTs and GNPs on the cementitious composite. This newly developed self-sensing cementitious composite exhibits potential for utility within the subgrade layer of road infrastructure.

## 2. Materials and Methods

### 2.1. Materials

**Figure 1:**
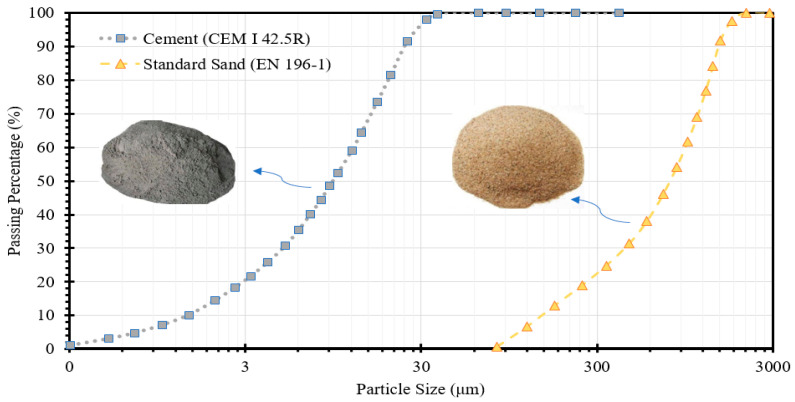
Particle size distribution.

The components employed in this study for the self-sensing cementitious composite consist of sand, cement, and a combination of conductive fillers, specifically multiwalled carbon nanotubes (MWCNTs) and graphene nanoplatelets (GNPs). In geotechnical analysis and the design of infrastructure projects, the particle size distributions of soils and stabilizers stands out as pivotal parameters demanding careful consideration. To this end, the particle size distributions (PSD) of the sand and cement used in this study can be found in Figure 1. It is noteworthy that for the present investigation, standard sand was utilized to minimize uncertainties regarding the electromechanical properties of the self-sensing cementitious composite arising from variations in matrix material gradation. However, for future research, the exploration of nonstandard sand is also recommended.

**Figure 2:**
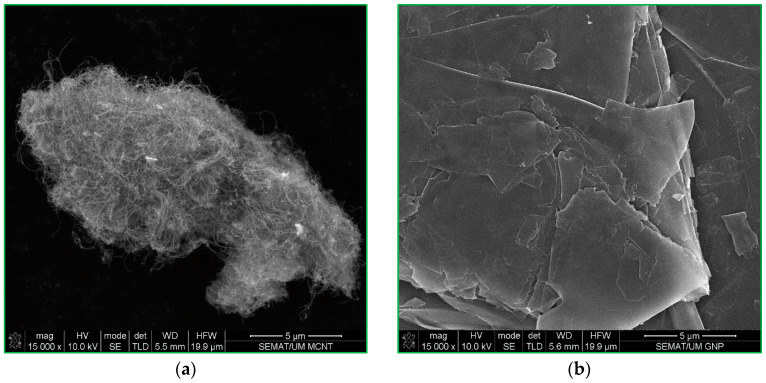
SEM images of (**a**) MWCNT and (**b**) GNP.

The choice of CEM I 42.5R cement was made as the stabilizing agent in this study due to its early high-strength properties. To optimize the impact of the conductive fillers on the electromechanical performance of the self-sensing cementitious composite and utilize them to their fullest advantage, a hybrid approach was adopted, as this issue has been proven in a previous study [26]. This involved the incorporation of both MWCNTs and GNPs into the cement-stabilized sand. Figure 2 exhibits the microstructure of these conductive fillers.

### 2.2. Mix Design

The composition ratios employed for the production of self-sensing cement-stabilized sand are presented in Table 1. Notably, Table 1 reveals that the self-sensing cementitious composite specimens used in this study exhibit varying concentrations of conductive fillers, ranging from 1% to 4%. In this research, a 10% cement content was employed for sand stabilization, a range consistent with that used in prior studies [1]. The challenge of agglomeration is a prominent concern in the manufacture of self-sensing cementitious composites. To address this issue, a hybrid dispersion technique was employed in this study. This technique involves the addition of 10% Pluronic F-127 by weight of conductive fillers as a surfactant, alongside bath sonication (CREST Ultrasonicator, CP 230 T), to ensure the effective dispersion of the MWCNT/GNP suspension, as outlined in [70]. Additionally, 50% TBP-97 by weight of the surfactant was introduced to mitigate foam formation. The specimen designation employed in Table 1 comprises two components: the abbreviation and the numeral. The abbreviation “SCS” represents self-sensing cement-stabilized sand, with the numeral indicating the percentage of MWCNT/GNP.

**Figure 3:**
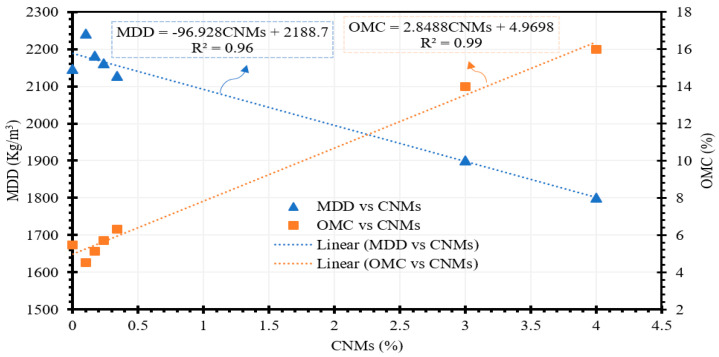
Correlation between maximum dry density (MDD) and carbon nanomaterials (CNMs); correlation between optimum moisture content (OMC) and carbon nanomaterials (CNMs).

The production of the specimens involves several sequential stages, including the preparation of well-dispersed conductive fillers following the methods described previously, the dry mixing of cement and sand, the integration of the well-dispersed conductive fillers with cement and sand to create the cementitious composite, and the molding of the specimens. The compaction characteristics of stabilized soils hold significant importance when molding the specimens [71,72]. Therefore, in this study, the maximum dry density (MDD) and optimum moisture content (OMC) were taken into consideration during the specimen fabrication process. Figure 3, presented in this context, illustrates the relationship between the OMC/MDD and the concentrations of MWCNTs/GNPs. As depicted in Figure 3, a clear trend emerges: MDD tends to decrease with an increase in carbon nanomaterials (CNMs), while OMC exhibits the opposite behavior, rising with increasing CNM concentration. This behavior is attributed to the high specific surface area of CNMs [73], which leads to water absorption, consequently elevating the OMC and lowering the MDD. With regard to the concentration of CNMs and its impact on the optimum moisture content (OMC) and maximum dry density (MDD), all specimens were produced in a cubic form with dimensions of 10 cm × 10 cm × 10 cm.

### 2.3. Piezoresistivity

**Figure 4:**
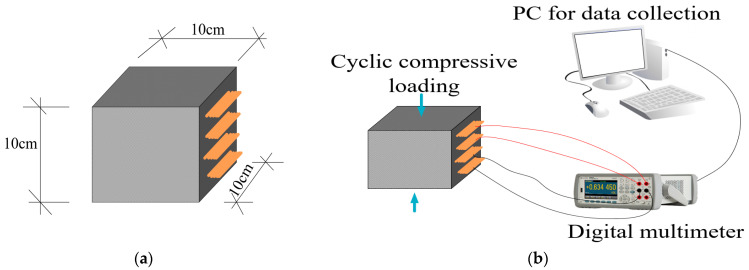
Schematic view of (**a**) specimen (**b**) electromechanical testing setup.

To assess the piezoresistive performance of the self-sensing cement-stabilized sand, after a 28-day curing process, the specimens were subjected to electromechanical testing. This testing involved the application of cyclic compressive loads using a loading machine (Lloyd 50kN) and the simultaneous recording of electrical resistance using a digital multimeter (Agilent 34461A 6½). To enhance the reliability of electrical resistance measurements, a four-electrode system was employed in the present study to mitigate the electrode contact resistance. Moreover, a resting period was incorporated before initiating the electromechanical testing to reduce polarization effects. Once a stable electrical resistance signal was achieved, electromechanical testing was initiated. A schematic representation of the specimens and the electromechanical testing setup is depicted in Figure 4.

In this research, a digital multimeter was utilized for the direct measurement of electrical resistance, eliminating the calculation stage for resistance measurement based on voltage and current. Subsequently, the recorded electrical resistance data were employed in the determination of electrical resistivity and the calculation of fractional changes in resistance or resistivity (FCR), as per Equations (1) and (2).

(1) $$\rho =\frac{RA}{L}$$

where ρ (Ω·cm), *A* (cm^2^), and *L* (cm) are the electrical resistivity, cross-sectional area, and distance between electrodes, respectively.

(2) $$FCR=\frac{∆R}{R}=\frac{∆\rho }{\rho }$$

where Δ*R* (Ω) is the change in resistance, *R* (Ω) is the initial resistance, ∆ρ (Ω·cm) is the change in resistivity, and ρ (Ω·cm) is the initial resistivity.

### 2.4. Electrochemical Impedance Spectroscopy (EIS)

**Figure 5:**
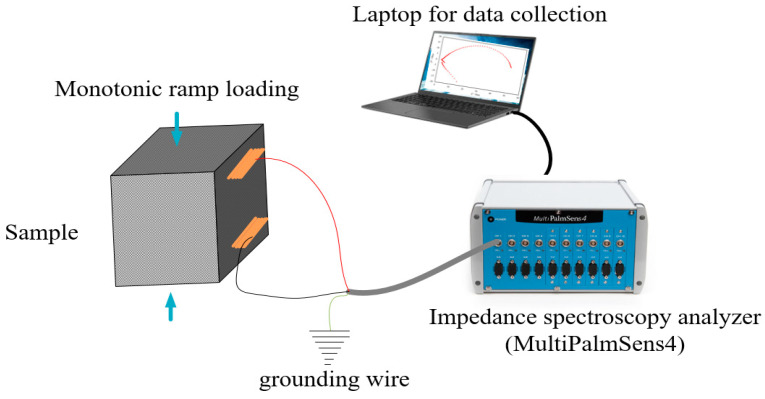
Testing setup for EIS analysis.

The sensing capability of self-sensing cementitious composites relies on creating conductive pathways by incorporating conductive fillers. Consequently, alterations in the microstructure of self-sensing cementitious composites lead to modifications in the state of these conductive pathways, resulting in changes in the impedance of the self-sensing cementitious composites. To assess the electromechanical performance of self-sensing cementitious composites with respect to their microstructural components and their corresponding variations, electrochemical impedance spectroscopy (EIS) can be employed. To achieve this objective, EIS measurements were conducted during the application of monotonic ramp loading, as illustrated in Figure 5. The impedance data were collected using an impedance analyzer (MultiPalmSens4, manufactured by PalmSens Bv) under various compressive stress levels, including 0 kPa, 320 kPa, 1276 kPa, 3188 kPa, and post-failure conditions. In the context of electrochemical impedance spectroscopy (EIS) analysis, a two-electrode measurement system was employed, a choice consistent with prior research that has validated the dependability of EIS outcomes using this specific two-electrode configuration [74].

In a direct current (DC) measurement system, Ohm’s law is the foundational principle that delineates the connection between voltage, current, and resistance, as articulated in Equation (3). Conversely, within an alternating current (AC) measurement system, the input voltage and output current exhibit time-dependent characteristics. Consequently, the inter-relationship between voltage, current, and impedance is governed by Equation (4). The application of Equation (5) consequently facilitates computation for the AC impedance spectroscopy.

(3) V=I×R

(4) $$V\left(t\right)=I(t)\times Z(\omega )$$

(5) Zω=V(t)I(t)=V0sin(ωt)I0sin⁡(ωt+φ)

In the given context, *V* (V) represents voltage, *I* (A) represents current, *R* (Ω) represents resistance, and *V*(*t*) and *I*(*t*) denote the time-varying voltage and current, respectively. The impedance is represented as *Z*(*ω*) (Ω), and the phase angle/phase change is denoted as φ (degree).

**Figure 6:**
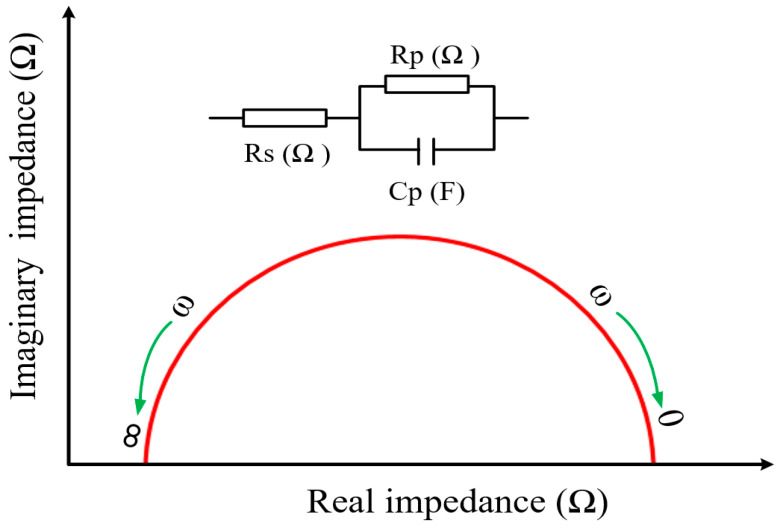
Schematic view of the Nyquist plot.

The Nyquist plot is a commonly used plot for evaluating microstructural changes in cementitious composites based on EIS measurements. Figure 6 illustrates a single Nyquist plot featuring resistance (R_s_, R_p_) and capacitance (C_p_) elements in a semicircular shape, commonly called a capacitive loop. Within the schematic model depicted in Figure 6 for a cementitious material, the symbol R_s_ represents the combined resistance of the solid and liquid phases, while R_p_ and C_p_ depict the interfacial resistance and capacitance associated with the interface between the solid and liquid phases, respectively [75]. The Nyquist plot depicts the relationship between the real impedance (*Z*′) and the imaginary impedance (*Z*″) across a range of frequencies. It is important to emphasize that the configuration of the Nyquist plot can exhibit various shapes and quantities, contingent upon the components and microstructural state of cementitious composites. Consequently, there is no universally accepted equivalent circuit model for cementitious composites, and multiple equivalent circuits are available for characterizing cementitious composites.

The Bode plot, which exhibits the connection between the magnitude of the absolute impedance (|*Z*|) and frequency (f), is another significant area of interest for examining the characteristics of cementitious composites. The magnitude of the absolute impedance can be determined using Equation (6):

(6) $$\left|Z\right|=\sqrt{{\left(Z^{\prime }\right)}^{2}+({Z^{\prime \prime })}^{2}}$$

### 2.5. Scanning Electron Microscopy (SEM)

To examine the microstructural attributes—including aspects such as form, dimensions, surface quality, and microscopic voids—a scanning electron microscopy (SEM-FEG, Nano SEM NOVA 200, FEI)investigation was undertaken on self-sensing cementitious composites. Within this experimental context, secondary electrons generated by an electron beam were directed onto the specimen’s surface, yielding three-dimensional visual representations. It is important to emphasize that specimens subjected to SEM analysis must undergo thorough drying prior to examination.

## 3. Results and Discussion

### 3.1. Influence of Conductive Filler Concentration on Electrical Resistivity

**Figure 7:**
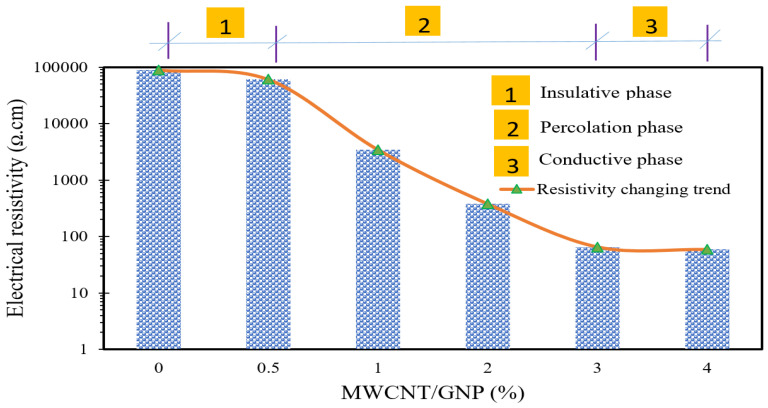
Impact of MWCNT/GNP concentration on electrical resistivity.

To achieve a well-sensitive self-sensing cementitious composite, the conductive filler concentration should reach beyond the percolation threshold. The percolation threshold signifies the proportion of conductive fillers at which there is a sudden decrease in electrical resistivity, thereby considerably enhancing the electrical conductivity of the cementitious composite. This relationship is graphically illustrated in Figure 7, depicting the correlation between electrical resistivity and concentration of multiwalled carbon nanotubes (MWCNTs) and graphene nanoplatelets (GNPs). Upon evaluating Figure 7, it becomes evident that the introduction of 0.5% MWCNTs/GNPs to the cementitious composite results in an insignificant change in electrical resistivity, indicating an insulating phase. During this insulating phase, the incorporation of carbon nanomaterials fails to establish conductive pathways, resulting in high electrical resistivity and diminished sensitivity of the cementitious composites. In this phase, the conduction mechanism relies solely on ionic conduction, as electronic conduction is absent due to the substantial separation between conductive fillers. Consequently, the sensitivity of self-sensing cementitious composites varies depending on environmental conditions, namely wet and dry conditions. In this insulating phase, the self-sensing cementitious composite may exhibit marginal sensitivity in wet conditions due to the movement of ions within the pores. Conversely, in dry conditions, ion movement within the pores is absent, leading to a lack of sensitivity. Hence, it is crucial to emphasize that exceeding the percolation threshold in terms of conductive filler concentration holds significant importance in achieving a highly sensitive cementitious composite under both dry and wet conditions.

As depicted in Figure 7, the intermediate stage spanning from 0.5% to 3% MWCNT/GNP concentration represents a transitional phase or percolation region. In this phase, the conductive mechanism is primarily driven by ionic conduction, the tunneling effect, and, towards the latter stages of this phase, direct contact conduction mechanisms. Consequently, well-defined conductive pathways are established. During this period, the separation between conductive fillers is relatively small, giving rise to localized conductive pathways. Due to the small distance between conductive fillers in this phase, electron conduction is predominantly facilitated through tunneling effects and direct contact conduction mechanisms. Consequently, variations in moisture content may not yield substantial alterations in electrical signals.

The final stage can be described as the conductive phase, in which the addition of conductive fillers leads to a minimal reduction in the electrical resistivity of the self-sensing cementitious composite, as illustrated in Figure 7. In this phase, the distance between the conductive fillers is very short, facilitating the conduction of electrons through tunneling effects, and, notably, via direct conduction mechanisms. Consequently, the absence of ion conduction does not impact the sensitivity of the self-sensing cementitious composite. Furthermore, even in this phase, an increase in moisture content results in an elevation of the electrical resistivity of the self-sensing cementitious composite. In summary, it is crucial to ensure that the concentration of conductive fillers reaches beyond a certain threshold (the start point of the percolation region) to achieve a well-established self-sensing cementitious composite.

### 3.2. Piezoresistivity

**Figure 8:**
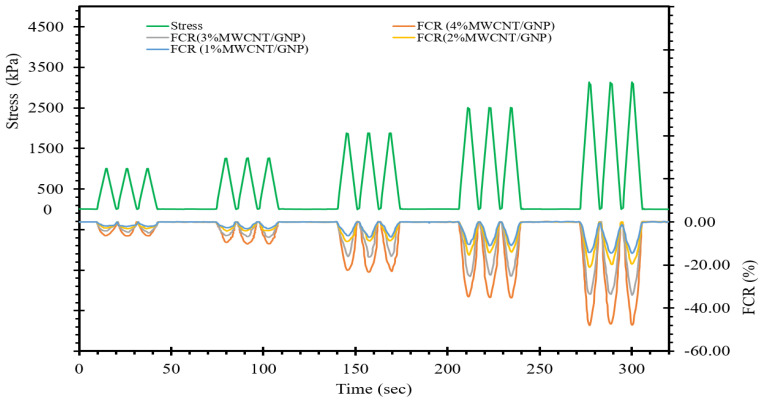
Stress/FCR-time.

Structural integrity assessment of transportation subgrades through structural health monitoring (SHM) offers valuable insights into the safety and dependability of structures, enabling the formulation of preemptive measures to preserve structural integrity in advance of critical damage. Within this framework, we delve into the piezoresistive characteristics of self-sensing cement-stabilized sand with the objective of offering valuable insights through its inherent stress and strain-sensing capability. In the preceding section, we examined the impact of conductive fillers on electrical resistance. To investigate this further, we subjected samples containing varying percentages of conductive fillers (ranging from 1% to 4%) to compressive cyclic loading. Figure 8 displays the fractional changes in resistance/resistivity (FCR) relative to cyclic stress over time. The results presented in Figure 8 clearly demonstrate that the FCR increases during compressive cyclic loading and decreases during unloading. The negative sign for FCR signifies the compressive nature of the loading process.

During compressive loading, the distance between conductive fillers diminishes, leading to an increase in FCR. Conversely, during unloading, the distance between conductive fillers increases, resulting in a reduction in the FCR. It is worth noting that the extent to which the initial position of conductive fillers is restored depends on the stress level. In other words, when the stress level falls within the quasielastic zone of the self-sensing cementitious composite, the initial position of the conductive fillers is almost completely regained, resulting in a zero FCR after each cyclic unloading. In this research, we tried to maintain the stress level within the quasielastic zone of the self-sensing cementitious composite, as discussed in a previously published paper that addressed the damage detection capability of this innovative material [15]. As a result, by considering the stress level within the quasielastic zone of this material, Figure 8 illustrates that the FCR returns to its initial value after each unloading cycle. Furthermore, Figure 8 highlights the high repeatability of the FCR fluctuations following multiple compressive cyclic loading events.

Furthermore, as depicted in Figure 8, it becomes apparent that at an equivalent level of cyclic compressive stress, the variation in the FCR becomes more pronounced as the proportion of MWCNTs/GNPs increases. This phenomenon signifies an enhancement in the self-sensing ability of the material with increasing concentration of conductive fillers. Incorporating a higher concentration of conductive fillers into cement-stabilized sand leads to an increase in the conductive pathways within the self-sensing cementitious composite. As illustrated in Figure 8, increasing the percentage of conductive fillers augments the sensitivity of the self-sensing cementitious composite due to the abundance of conductive pathways. In summary, the stress-sensing capacity of the self-sensing cementitious composite exhibits an upwards trend in response to the escalation of conductive filler concentration.

**Figure 9:**
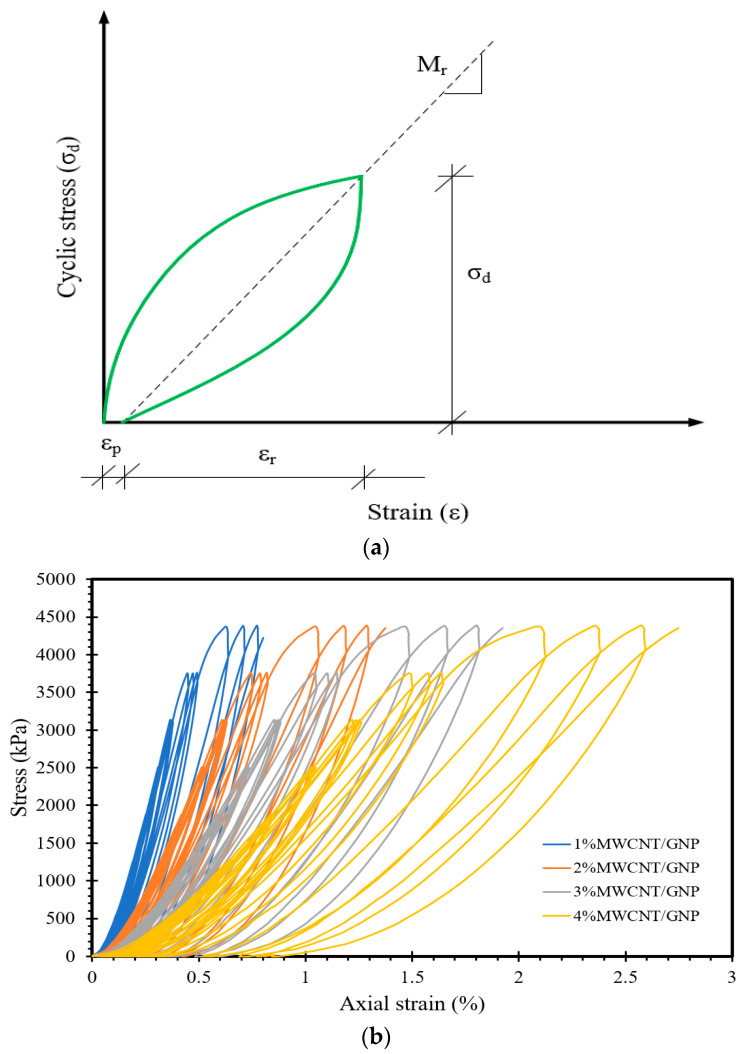
(**a**) Determination of the resilient modulus (**b**) stress–strain for the self-sensing cementitious composites.

The resilient modulus (Mr) of the subgrade, as determined through Equation (7), constitutes a crucial factor in pavement design. Hence, it is essential to investigate the influence of MWCNTs/GNPs on the resilient modulus of stabilized sand, as well as the relationship between the resilient modulus and FCR over time. Figure 9a provides a visual representation of the concept of resilient modulus, as defined by cyclic deviator stress and resilient strain. Consequently, Figure 9b displays the cyclic stress–strain characteristics of the self-sensing cementitious composites, which were employed to calculate the resilient modulus.

(7) $$M_{r}=\frac{\sigma_{d}}{\varepsilon_{r}}$$

**Figure 10:**
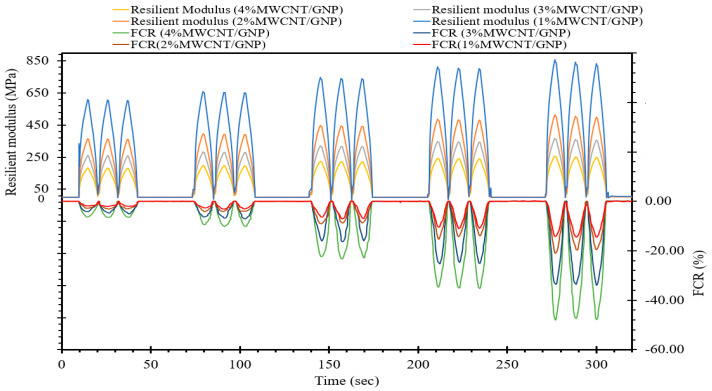
Resilient modulus/FCR-time.

Considering the procedure outlined in Figure 9, we calculated the resilient modulus of self-sensing cementitious composites and depicted its relationship with the FCR over time in Figure 10. As depicted in Figure 10, it becomes evident that over time, the FCR of self-sensing cementitious composites containing various MWCNTs/GNPs increases and decreases with increasing and decreasing resilient modulus, respectively. It should be noted that the cyclic stress levels employed in this study to assess stress/strain-sensing behavior fall within the quasi-elastic range of the self-sensing cementitious composite. This condition (i.e., stress level within the quasi-elastic region) leads to an increase in the resilient modulus with increasing deviator stress level, resulting in an augmentation of the FCR variations with an increasing resilient modulus over time, as shown in Figure 10. A comparable pattern (an increase in the resilient modulus with rising deviator stress) was also documented in a prior research investigation [76]. However, in certain instances, when the resilient strain increases in response to elevated stress levels, the resilient modulus exhibits a reduction with increasing deviator stress levels [77].

Furthermore, Figure 10 provides insights into the relationship between the resilient modulus and the concentration of MWCNTs/GNPs. As observed in Figure 10, it becomes apparent that the resilient modulus decreases with increasing percentage of MWCNTs/GNPs. This diminishing trend for the resilient modulus with increasing MWCNT/GNP concentration can be attributed to two key factors: the hindrance of cement hydration by carbon nanomaterials and the influence of moisture content.

Carbon nanomaterials have the capacity to localize themselves between cement particles, impeding the cement hydration process and rendering the specimens more ductile. Another factor contributing to the diminishing trend for the resilient modulus with increasing MWCNT/GNP content is the rising moisture content, which is evident from the discussion for Figure 3. The optimal moisture content selected for specimen preparation displays an increasing tendency with increasing MWCNT/GNP percentage. Consequently, the growing moisture content with increasing MWCNT/GNP ratio serves as another factor that leads to a reduction in the resilient modulus [77]. Matric suction in soil represents another critical element necessitating inclusion for assessing the geotechnical characteristics of natural and stabilized soil [78]. The resilient modulus of soils exhibits an upwards trend with increasing matric suction levels [79]. Consequently, the decrease in matric suction due to an increase in moisture content is an additional contributing factor that contributes to the reduction in resilient modulus with increasing MWCNT/GNP levels. It is worth noting that lower concentrations of MWCNTs/GNPs can enhance the resilient modulus of cementitious composites. However, in the current study, the higher percentage variations are mainly considered for evaluating the nonstructural properties of self-sensing cementitious composites (stress/strain and damage sensing capability) since a percentage that is lower than the percolation threshold is ineffective for this purpose. Furthermore, despite the decrease in the resilient modulus with increasing proportion of MWCNTs/GNPs, it is noteworthy that the obtained values for the resilient modulus surpass the resilient modulus values reported in previous studies for different soils [80,81,82,83,84].

Given that these two factors (the hindrance of cement hydration by carbon nanomaterials and the influence of moisture content) collectively lead to a reduction in the resilient modulus with increasing MWCNT/GNP, it can be concluded that the stiffness of self-sensing cementitious composites diminishes with a higher presence of carbon nanomaterials. This phenomenon is clearly discernible in Figure 10, in which a higher peak value for the resilient modulus corresponds to a lower peak in the FCR. For example, Figure 10 demonstrates that in specific instances the FCR variations in self-sensing specimens containing 1% MWCNTs/GNPs are lower than those in specimens containing 2%, 3%, and 4% MWCNTs/GNPs due to the higher resilient modulus. A similar trend is apparent when comparing other specimens. This trend is directly linked to the stiffness of self-sensing cementitious composites. The variations in the FCR are contingent upon the alterations in conductive pathways formed by conductive fillers. Consequently, when self-sensing cementitious composites exhibit greater stiffness (higher resilient modulus), the variations in conductive pathways within these composites are less pronounced, resulting in a reduced fluctuation in the FCR under cyclic loading.

**Figure 11:**
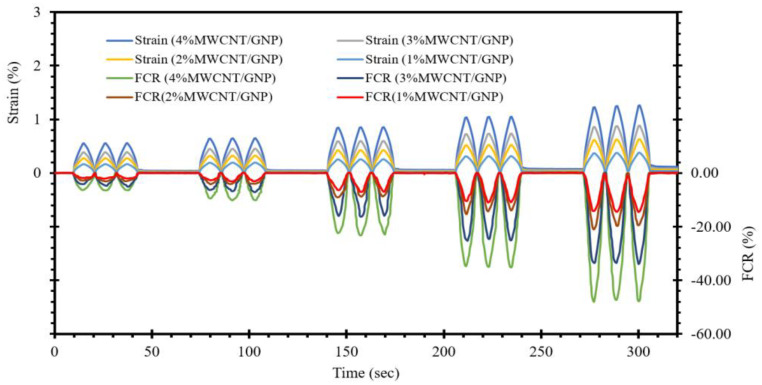
Strain/FCR-time.

Figure 11 displays the strain detection capabilities of a self-sensing cementitious composite when subjected to repetitive loading within the quasielastic range. The strain increases as the cyclic compressive load intensifies, leading to an increase in the FCR changes, as indicated in Figure 11. This increased cyclic compressive loading disrupts the microstructures and conductive pathways, increasing the FCR changes. Nonetheless, the cyclic compressive loading applied within the quasielastic range induces quasielastic strain, allowing the strain to recover after each unloading step, thereby preventing any drift. It is worth noting that this study focuses solely on the strain-sensing performance of the self-sensing cementitious composite, while an evaluation of damage sensing can be found in a previously published study. Furthermore, as depicted in Figure 11, it becomes apparent that the fluctuation in the FCR (fractional change in resistance) during cyclic strain shows a noticeable increase with increasing MWCNT/GNP content percentage. This increase in MWCNT/GNP content gives rise to amplification in conductive pathways, thereby enhancing strain-sensing capabilities.

### 3.3. Electrochemical Impedance Spectroscopy (EIS)

**Figure 12:**
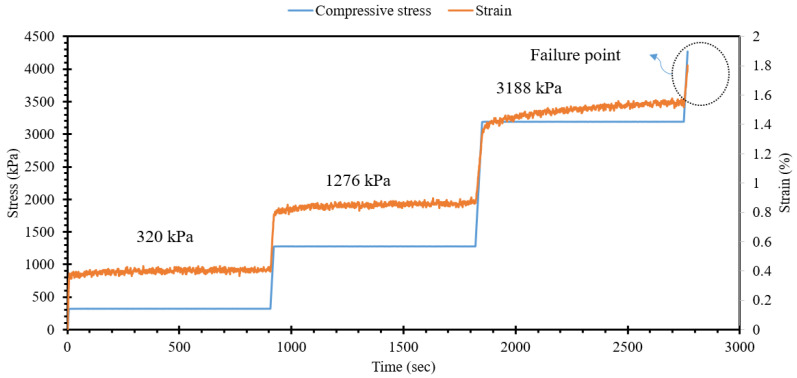
Compressive ramp loading.

Electrochemical impedance spectroscopy (EIS) is a nondestructive technique extensively employed for assessing the electrochemical characteristics of cementitious composites. EIS proves to be a valuable methodology for discerning alterations in the microstructure of cementitious composites induced by a variety of factors, including curing conditions, types of cementitious materials, fluctuations in moisture content, variations in temperature, and changes in fabric composition [61,64,74,85]. Consequently, it is evident that EIS can be effectively utilized to appraise the impact of external loading on the deformation of the microstructure in self-sensing cementitious composites. To this end, a self-sensing cementitious composite incorporating 3% MWCNTs/GNPs was subjected to a stress path, as depicted in Figure 12, to investigate the EIS response under different stress levels. The EIS data were collected at stress levels of 0 kPa, 320 kPa, 1276 kPa, and 3188 kPa and beyond the point of structural failure, as illustrated in Figure 12.

**Figure 13:**
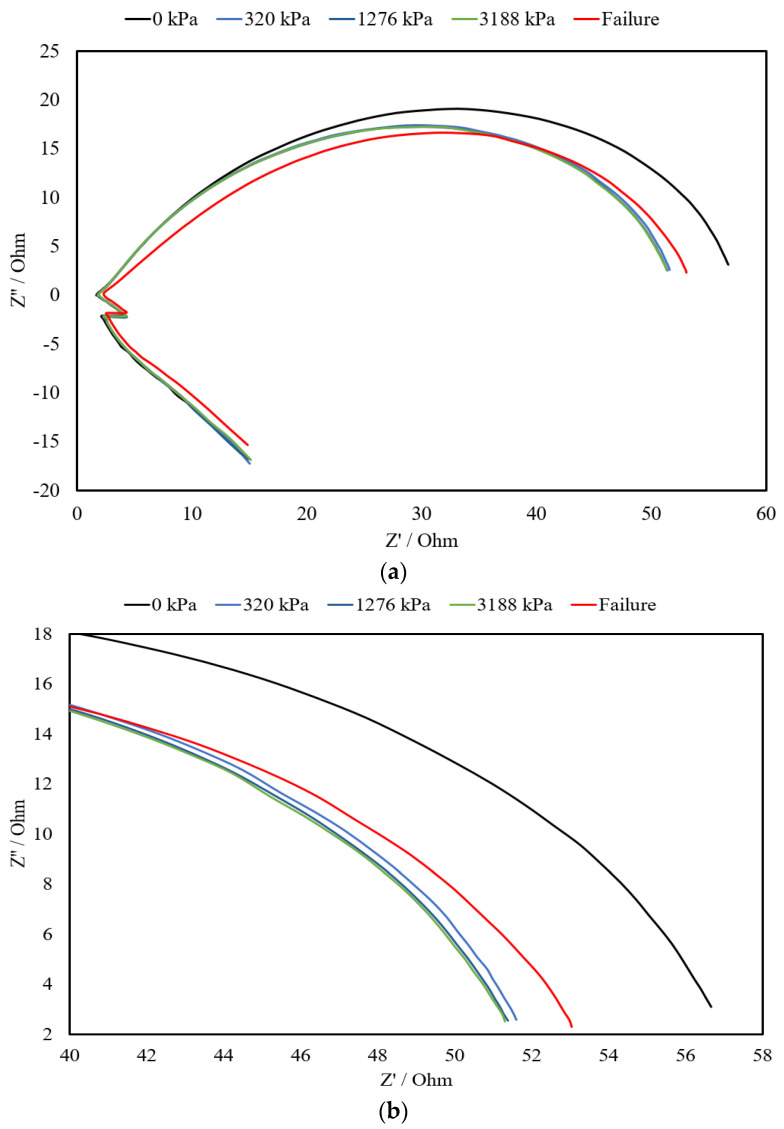
Electrochemical impedance spectroscopy (EIS) for specimens containing 3% CNMs under the compressive ramp loading pattern shown in Figure (12). (**a**) The complete EIS curve; (**b**) the zoomed view.

Figure 13 depicts the Nyquist plots obtained for a self-sensing cementitious composite containing 3% MWCNTs/GNPs, subject to the abovementioned stress levels. In reference to Figure 13a, it is evident that the diameter of the semicircular curve diminishes when exposed to compressive ramp loading. In simpler terms, the application of ramp loading reduces the gaps between conductive fillers and enhances the density of conductive pathways, resulting in a decrease in electrical impedance. Consequently, the diameter of the semicircular curves decreases with increasing stress levels due to the contraction of micropores and spaces, as explicitly highlighted in the magnified section of Figure 13b. However, upon failure (cracks appear on the surface of the specimen), microcracks and pores become apparent and expand, leading to an enlargement of the semicircular curve, as indicated by the red curve in Figure 13b. Therefore, it can be affirmed that the fluctuations observed in the Nyquist plots during stress testing offer valuable insights into the microstructural state of self-sensing cementitious composites. In general, the findings presented herein align with those of a prior investigation in which the impact of cracks, healing, and the duration of the curing process were examined regarding the variations observed for electrochemical impedance spectroscopy (EIS) carried out for a conventional cementitious composite [59]. However, the different shapes and changing trends for the Nyquist plots found in this study compared to those in a previous study [59] might be due to the MWCNT/GNP conductive fillers used in the current study.

### 3.4. Microstructure Analysis

**Figure 14:**
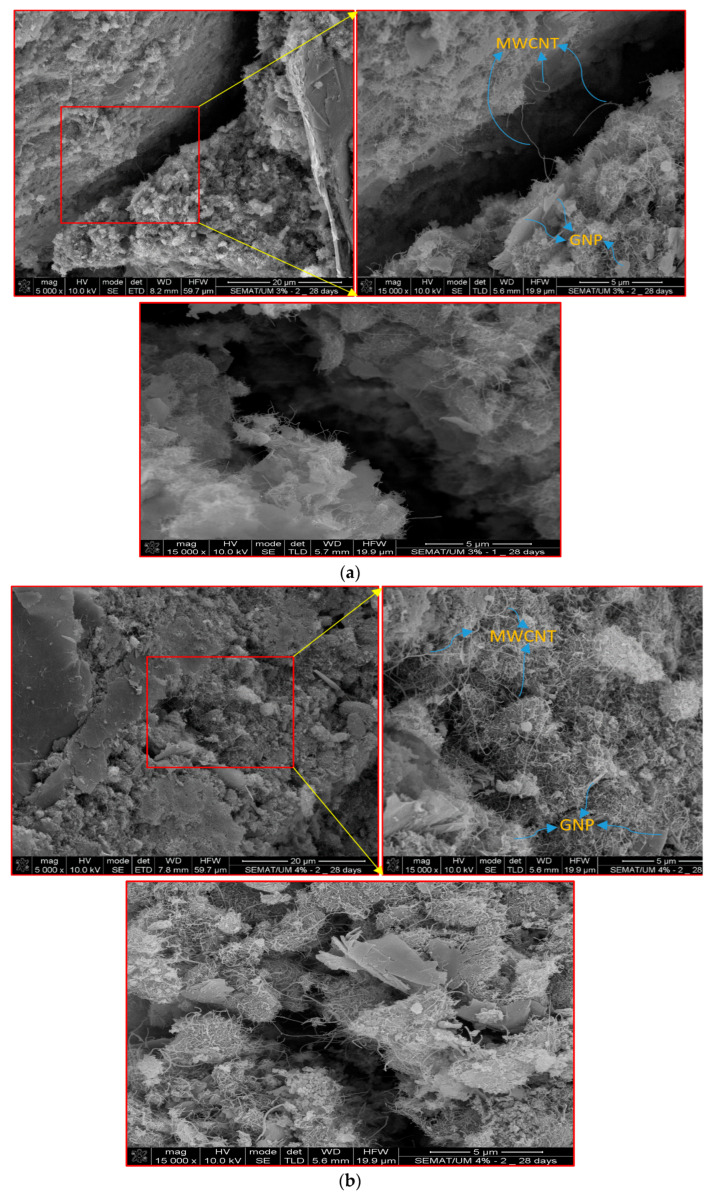
Morphology of self-sensing cementitious composites containing (**a**) 3% MWCNTs/GNP and (**b**) 4% MWCNTs/GNP.

Figure 14 presents the microstructural characteristics of self-sensing cementitious composites incorporating 3% MWCNTs/GNPs and 4% MWCNTs/GNPs. As depicted in Figure 14, it is discernible that the congestion of carbon nanomaterials (CNMs) increases as the percentage of conductive fillers rises, consequently increasing the sensitivity due to an augmented network of conductive pathways, as explained in the context of the stress/strain-sensing performance of this novel self-sensing cementitious composite.

As shown in Figure 14a, the bridging effects of MWCNTs are evident, connecting two sides of a microspace within the specimen containing 3% MWCNTs/GNPs. This bridging effect contributes to the sensory capabilities exhibited by the self-sensing cementitious composite. On the other hand, Figure 14b illustrates the overlapping arrangement of MWCNTs, where they intersect with each other in the self-sensing cementitious composite containing 4% MWCNTs/GNPs. This configuration enhances the composite’s stress/strain-sensitivity performance more in comparison to the self-sensing cementitious composite containing 3% MWCNTs/GNPs. Additionally, the presence of graphene nanoplatelets (GNPs) is noticeable in both Figure 14a,b, serving to fill microgaps within the structure.

Generally, the mechanical strength of cementitious composites increases proportionally with the addition of carbon nanomaterials, especially up to a specific threshold, notably within very small percentages [86]. However, it is essential to note that cementitious composites with low concentrations of carbon nanomaterials do not exhibit notable sensing performance. To achieve optimal sensing capabilities in self-sensing cementitious composites, the concentration of conductive fillers must fall within the percolation threshold [87]. In this context, a compromise between the mechanical and electromechanical properties of self-sensing cementitious composites may arise due to the agglomeration of carbon nanomaterials, as depicted in Figure 14b. The likelihood of agglomeration rises with an increased concentration of conductive fillers. With reference to Figure 14, it is evident that dispersion quality is significant in specimens doped with 3% MWCNT/GNP, whereas specimen doped with 4% MWCNT/GNP displays bundled carbon nanomaterials on the surface of SEM images. Therefore, while the self-sensing cement-stabilized sand doped with 4% MWCNT/GNP exhibits higher sensitivity, it is crucial to take into account the adverse impacts of agglomeration on mechanical strength. In this context, the concentration of 3% MWCNT/GNP can be identified as the optimal level, as it also represents the upper limit of the percolation threshold, as illustrated in Figure 7.

## 4. Conclusions

The present investigation delves into the evaluation of stress- and strain-sensing capabilities as well as damage detection performance of self-sensing cementitious composites. This assessment was conducted utilizing piezoresistivity and electrochemical impedance spectroscopy (EIS) measurement systems, employing a four-probe system for piezoresistivity analysis and a two-probe system for EIS analysis. In summary, the following key findings and conclusions can be drawn from this study:

1. In the present research, a hybrid dispersion method involving chemical treatment and ultrasonication was utilized to attain a homogeneous dispersion of conductive fillers within a self-sensing cementitious composite.
2. The stress- and strain-sensing capabilities exhibit an upwards trend as the concentration of MWCNTs/GNPs is increased and as the applied stress levels are intensified. However, it is crucial to underscore that the stress levels used in this study to assess the stress and strain-sensing performance are confined within the quasi-elastic region, as established by the findings of our previous study [15].
3. The resilient modulus exhibits a declining trend with increasing percentage of MWCNTs/GNPs, signifying a reduction in stiffness in correlation with the increased MWCNT/GNP content. For example, when the self-sensing cementitious geocomposite underwent cyclic compressive stress equivalent to 1000 kPa, the resilient modulus decreased from 605 MPa to 365 MPa, 260 MPa, and 180 MPa, respectively, with an increase in MWCNTs/GNPs from 1% to 2%, 3%, and 4%. Detailed information on the reduction of resilient modulus with the escalating concentration of MWCNTs/GNPs under various levels of cyclic compressive stress can be evaluated in Figure 10. This reduced stiffness is observed to be associated with an augmented peak value for the FCR variation, which is attributed to a more pronounced variability in the conductive pathways formed by the conductive fillers.
4. An assessment of the damage detection capacity for a self-sensing cementitious composite with a 3% MWCNT/GNP composition was conducted through electrochemical impedance spectroscopy (EIS) analysis. The findings reveal a reduction in the dimension of the Nyquist plots during the application of a compressive load prior to structural failure. Conversely, upon failure, the Nyquist plots exhibit an expansion. The decrease in the Nyquist plot size can be ascribed to the diminishing distances between conductive fillers. In contrast, the enlargement of the Nyquist plot is attributed to an increase in the separation between these conductive fillers.
5. Scanning electron microscopy (SEM) provides visual evidence for an accumulation of conductive pathways with increasing concentration of conductive fillers. The MWCNTs play a role in bridging microcracks and pore spaces, while the GNPs fill the voids. Consequently, incorporating hybrid conductive fillers in cementitious composites offers a more efficient approach to inducing sensory (stress/strain and damage sensing) performances. SEM analysis reveals that to prevent the agglomeration phenomenon, it is crucial not only to employ effective dispersion techniques but also to maintain the concentration of conductive fillers within the percolation threshold.

This investigation explored the stress/strain-sensing and damage detection capabilities of self-sensing cementitious composites incorporating hybrid conductive fillers (MWCNT/GNP) for possible application in subgrade of rigid pavement, flexible pavement, and railway track. While piezoresistivity has been extensively studied in the past, it is worth noting that there is a need for further exploration in future research studies, particularly concerning the EIS analysis of self-sensing cementitious composites as a pavement construction material.

## Figures and Tables

**Table 1:** Mix design of self-sensing cement-stabilized sand.

Designation	MWCNT/GNP (%)	Cement (% by Weight of Dry Sand)	Pluronic F-127 (% by Weight of MWCNT/GNP)	TBP-97 (% by Weight of Surfactant)
SCS1	1	10	10	50
SCS2	2	10	10	50
SCS3	3	10	10	50
SCS4	4	10	10	50

## Data Availability

Data are contained within the article.
